# Acute Hemolysis with Renal Failure due to* Clostridium* Bacteremia in a Patient with AML

**DOI:** 10.1155/2016/6549268

**Published:** 2016-09-27

**Authors:** R. M. Medrano-Juarez, D. Sotello, M. A. Orellana-Barrios, L. D'Cuhna, J. D. Payne, K. Nugent

**Affiliations:** Department of Internal Medicine, Texas Tech University Health Sciences Center, 3601 4th Street, MS 9410, Lubbock, TX 79430, USA

## Abstract

We present a case of acute hemolytic anemia, renal failure, and* Clostridium perfringens* bacteremia in a patient with acute myelogenous leukemia. The high fatality of* C. perfringens* bacteremia requires that clinicians recognize and rapidly treat patients at risk for this infection. Although other hemolytic processes are in the differential diagnosis of these events, the presence of high fever, chills, and rapidly positive blood cultures may help narrow the diagnosis. Most cases of* C. perfringens* bacteremia have a concomitant coinfection, which makes broad spectrum empiric therapy essential. There is a high mortality rate of* C. perfringens* infections associated with leukemia.

## 1. Introduction

Patients with hematologic malignancies are at particularly high risk of aggressive and sometimes fatal infections. Prompt diagnosis may be clinically challenging with concomitant or differential diagnoses such as disseminated intravascular coagulation, hemolysis, tumor lysis syndrome, and thrombotic thrombocytopenic purpura. We present a case of acute hemolytic anemia, renal failure, and* Clostridium perfringens* bacteremia in a patient with acute myelogenous leukemia.

## 2. Case Presentation

A 32-year-old African American man initially presented with a three-day history of gingival hemorrhage, occurring after tooth extraction. He had a past medical history of possible lead poisoning as a child, hypertension, and a three-year history of thrombocytopenia presumably due to idiopathic thrombocytopenic purpura. At presentation, he was not taking any medications. He was diagnosed with acute myeloid leukemia (karyotype 47, XY + 21). He failed initial induction with cytarabine/daunorubicin and subsequently underwent reinduction with cytarabine/clofarabine.

Due to sustained severe neutropenia and fever during his inductions, he completed 25 days of empiric treatment with meropenem. Ultimately, he developed vancomycin-resistant* Enterococcus faecium* gluteal abscess, treated with incision and drainage and 15 days of intravenous (IV) quinupristin/dalfopristin. He then received prophylactic ciprofloxacin/fluconazole.

Approximately seven days later his gluteal wound had almost healed, and he had been afebrile for >10 days. His absolute neutrophil count remained extremely low (persistently < 10 cells K/uL). The patient developed sudden onset chills, body aches, fever, diaphoresis, and dark urine. Physical examination presented a patient in severe distress, with pale mucosae and mild mucositis, blood pressure 134/84 mmHg, heart rate 126 beats per minute, respirations 20 per minute, room air oxygen saturation 94%, icteric sclera, no lymphadenopathy, clear lung sounds, normal S1 and S2 with a systolic aortic 2/6 murmur, normal bowel sounds, slight abdominal distention with no tenderness, no hepatomegaly, 1.5 cm almost healed perianal/gluteal ulcer with no secretions or crepitus, normal genitals, mild bilateral pretibial edema, unremarkable chemotherapy port site, and no abnormal neurological findings. The patient's laboratory results differed significantly from the previous day (Table 1). Notably, he had evidence of acute hemolytic anemia and acute renal failure.

He was again started on empiric antibiotics, including IV meropenem, levofloxacin, daptomycin, and voriconazole. Peripheral blood smear showed severe pancytopenia with some schistocytes. Blood cultures were drawn, and within 12 hours Gram stain of blood culture medium showed Gram-positive rods. Final growth was reported 24 hours later as* Clostridium perfringens *and* Enterococcus faecalis* (vanA and vanB negative). Antibiotics were adjusted to IV penicillin G and daptomycin, which he continued for 8 days. His hemolysis, renal failure, and fever improved within 48 hours, but his ANC was persistently low despite filgrastim therapy. He required temporary hemodialysis due to renal failure but recovered renal function within eight weeks. The patient's bacteremia was resolved within 48 hours. Approximately 12 weeks later, a follow-up peripheral blood smear showed persistent leukemic blasts. At this point, the patient decided against further treatment and opted for hospice care.

## 3. Discussion

*Clostridium *spp. are anaerobic Gram-positive rods capable of forming endospores which make them resilient in harsh environmental conditions and ubiquitous in nature. Although* Clostridium *spp. constitute normal gastrointestinal or genital flora, several clostridial species are associated with human disease either by direct tissue infection (e.g.,* C. perfringens* and* C. septicum*) or by toxin production (e.g.,* C. difficile*,* C. botulinum*, and* C. tetani*) [1].

*C. perfringens* is classically associated with myonecrosis, or gas gangrene, and food poisoning. The pathophysiology of myonecrosis involves several exotoxins, including lecithinase, an alpha-toxin with phospholipase activity [2]. Acute hemolysis due to this alpha-toxin has been reported in up to 15% of patients with* C. perfringens* bacteremia [3]. Predisposing risk factors for* C. perfringens* infections include renal failure [4], diabetes [5], inflammatory bowel disease [6], diverticulitis and appendicitis [7, 8], gastrointestinal surgery [9], solid tumors [10], stem cell transplant [11, 12], lymphoma [13], and leukemia [14, 15].

As seen in our patient,* C. perfringens* bacteremia in patients with leukemia frequently occurs concomitant with other bacteria. For example, Bodey et al. [10] reported that 20 of 47* C. perfringens* bacteremia cases were polymicrobial. In this report, the response rate was lower in the polymicrobial group (30% versus 52%). We performed a PubMed search of adult cases of* C. perfringens* infections associated with leukemias. A total of 19 reported cases (7 female, 12 male) were found [3, 10, 11, 14–17], with a mean age of 47.5 years (see Table 2). Including our case, survival was estimated at 15%, demonstrating high fatality associated with* C. perfringens* infections.

Prompt recognition and rapid initiation of appropriate antibiotic therapy are essential to increase survival in* C. perfringens* infections, particularly in immunosuppressed patients. The rapid onset of systemic inflammatory response, hemolysis, and possibly renal failure should prompt the consideration of clostridial disease. The differential diagnosis includes hemolytic uremic syndrome and disseminated intravascular coagulation, which can occur in sepsis and in patients with lymphoproliferative disorders. The presence of high fever, bacteremia, rapid deterioration with hemolysis, renal failure, and shock may also provide clues to narrow the diagnosis. Empiric treatment, usually with penicillin, clindamycin, metronidazole, or cephalosporins, should be started without delay while awaiting cultures. There is some evidence that certain antibiotics, such as clindamycin and tetracycline, have greater ability to inhibit toxin synthesis, and this may produce a therapeutic benefit [18]. Surgical intervention and the combination of penicillin and clindamycin have also been reported as factors significantly associated with improved survival [13].

## 4. Conclusion

A high index of suspicion must be maintained for* C. perfringens* infection in patients at risk given the possibility of rapid deterioration with shock, acute massive hemolysis, and acute renal failure. Although other hemolytic processes are in the differential diagnosis of these events, the presence of high fever, chills, and rapidly positive blood cultures may help narrow the diagnosis. Most cases of* C. perfringens* bacteremia have a concomitant coinfection, which makes broad spectrum empiric therapy essential. There is a high mortality rate of* C. perfringens* infections associated with leukemia.

## Figures and Tables

**Table 1:** Laboratory values.

Parameter	Previous	Day of fever	Units
WBC	0.1	0.1	K/uL
RBC	2.92	1.63	M/uL
Hgb	8.6	5.2	g/dL
Hct	24.1	13.7	%
MCV	82.7	84.4	fL
MCH	29.4	31.9	pg
RDW	12.7	13.1	%
Platelet	16	11	K/uL
MPV	7.7	8.9	fL
Na	137	130	mmol/L
K	3.8	4.9	mmol/L
Cl	100	96	mmol/L
Mg	1.6	1.5	mg/dL
Ca	8.8	8	mg/dL
Ph	3.7	2	mg/dL
Bicarb	25	20	mmol/L
BUN	15	37	mg/dL
Creatinine	1.2	2.4	mg/dL
Uric acid		4.3	mg/dL
Total bilirubin	1.7	13.4	mg/dL
Direct bilirubin		8.3	mg/dL
LDH	120	1132	IU/L
ALT	97	130	IU/L
AST	167	36	IU/L
PT		18	seg
INR		1.6	
PTT		58.2	sec
Fibrinogen		517	mg/dL
D dimer		6200	ng/mL
Coombs		Positive	
Haptoglobin		<15	mg/dL
vWF protease activity		64	%

**Table 2:** 

Authors	Age	Sex	Leukemia type	Survival
Ifthikaruddin and Holmes [16]	54	F	AML	N
van Bunderen et al. [3]	73	F	CLL	N
Vaiopoulos et al. [15]	74	M	AML	N
Pirrotta et al. [14]	50	M	ALL	N
Kapoor et al. [17]	58	M	AML	N
Bodey et al. [10]	30	F	NSAL	N
	27	F	NSAL	N
	47	M	NSAL	Y
	64	M	NSAL	N
	53	F	NSAL	N
	58	F	NSAL	N
	50	M	NSAL	N
	37	M	NSAL	N
	20	M	NSAL	Y
	53	M	NSAL	N
	19	M	NSAL	N
	60	M	NSAL	N
	19	F	NSAL	N
Lee et al. [11]	57	M	ALL	N
Our case	32	M	AML	Y

AML: acute myelogenous leukemia; CLL: chronic lymphocytic leukemia; NSAL: nonspecified acute leukemia; ALL: acute lymphocytic leukemia; N: no survival; Y: survival.
